# α-Glucosidase Inhibition Mechanism and Anti-Hyperglycemic Effects of Flavonoids from Astragali Radix and Their Mixture Effects

**DOI:** 10.3390/ph18050744

**Published:** 2025-05-18

**Authors:** Xing Han, Pengpu Wang, Jing Zhang, Yang Lv, Zhigao Zhao, Fengxian Zhang, Mingying Shang, Guangxue Liu, Xuan Wang, Shaoqing Cai, Feng Xu

**Affiliations:** 1 State Key Laboratory of Natural and Biomimetic Drugs, School of Pharmaceutical Sciences, Peking University, No. 38 Xueyuan Road, Beijing 100191, China; 2011110096@stu.pku.edu.cn (X.H.); 20240025@immu.edu.cn (P.W.); 20241083@zcmu.edu.cn (J.Z.); victoryjack@pku.edu.cn (Y.L.); zhigaozhao@my.swjtu.edu.cn (Z.Z.); 20210931204@bucm.edu.cn (F.Z.); myshang@bjmu.edu.cn (M.S.); guangxl@bjmu.edu.cn (G.L.); 2 School of Public Health, Inner Mongolia Medical University, Hohhot 010110, China; 3 School of Pharmaceutical Sciences, Zhejiang Chinese Medical University, Hangzhou 310053, China; 4 College of Life Science and Engineering, Southwest Jiaotong University, Chengdu 610031, China; 5 School of Life Sciences, Beijing University of Chinese Medicine, Beijing 102488, China; 6 Department of Chemical Biology, School of Pharmaceutical Sciences, Peking University, No. 38 Xueyuan Road, Beijing 100191, China; xuanwang6818@bjmu.edu.cn; 7 Key Laboratory of State Administration of Traditional Chinese Medicine (TCM) for Compatibility Toxicology, Beijing 100191, China

**Keywords:** astragali radix, flavonoids, α-glucosidase, inhibitory mechanism, postprandial hyperglycemia, mixture effect

## Abstract

**Background**: Inhibition of intestinal α-glucosidase is a key strategy for controlling postprandial hyperglycemia in diabetes. Astragali Radix (AR), a traditional medicinal and dietary herb widely consumed in China, is rich in flavonoids that are believed to exhibit hypoglycemic properties. **Methods**: A total of 29 AR-related flavonoids, including both original constituents and metabolites, were screened for *α*-glucosidase inhibitory activity using in vitro enzymatic assays. Mechanistic investigations were conducted through enzyme kinetics, circular dichroism (CD) spectroscopy, surface plasmon resonance (SPR), and molecular docking. The in vivo hypoglycemic effects were assessed using a postprandial hyperglycemic mouse model. Additionally, potential mixture effects of flavonoid combinations were evaluated. **Results**: Of the 29 flavonoids, 16 demonstrated significant α-glucosidase inhibitory activity, with five (**C3**, **C17**, **C19**, **C28**, and **C29**) identified as novel inhibitors. Structure–activity relationship (SAR) analysis revealed that hydroxylation, particularly at the C-3 position, enhanced activity, while glycosylation and methoxylation reduced it. Mechanistic studies demonstrated that these compounds bind to distinct amino acid residues within the active site of α-glucosidase, inducing conformational changes and exerting different types of inhibition, leading to varying inhibitory mechanisms. Additionally, 15 compounds reduced postprandial blood glucose levels, with **C3**, **C16**, **C17**, **C19**, and **C28** confirmed as novel in vivo inhibitors. Notably, two compositions of flavonoids combined at their individually ineffective concentrations exhibited significant inhibitory effects. **Conclusions**: This study provides a comprehensive evaluation of AR-related flavonoids as α-glucosidase inhibitors and offers valuable insights for the development of highly effective, low-toxicity, flavonoid-based, antidiabetic therapeutics and functional foods.

## 1. Introduction

Diabetes mellitus (DM) is a chronic metabolic disorder characterized by persistent hyperglycemia. With improved living standards and changing lifestyles, the incidence of DM and its associated complications has risen significantly over the past few decades [1]. According to the International Diabetes Federation (IDF), approximately 537 million individuals worldwide were living with diabetes in 2021, accounting for 10.5% of the global population. This figure is projected to increase to 12.2% (783.2 million individuals) by 2045 [2]. Persistent hyperglycemia in DM can lead to severe complications, including blindness, kidney disease, neuropathy, and cardiovascular disorders [3]. To combat the rapidly increasing prevalence of DM, prevention and control strategies are urgently needed. Among these, the management of postprandial hyperglycemia, a key contributing factor in the onset and progression of DM, is critical. And α-glucosidase inhibitors have been recommended as a first-line treatment for reducing postprandial hyperglycemia and mitigating the progression of DM [4].

The α-glucosidase, a key glucoside hydrolase located on the brush border of small intestine cells, catalyzes the hydrolysis of disaccharides and oligosaccharides into absorbable monosaccharides, completing the final step of carbohydrate digestion [5]. α-Glucosidase inhibitors can delay carbohydrate digestion and glucose absorption, thereby reducing postprandial plasma glucose levels and providing an effective treatment for diabetes [6]. However, commonly used α-glucosidase inhibitors, such as acarbose, voglibose, and miglitol, are often associated with gastrointestinal side effects, including abdominal distension, diarrhea, and abdominal pain [6,7,8]. Modern pharmacological studies have demonstrated that natural compounds offer a promising alternative for delaying the onset of DM or mitigating disease complications in its early stages. Thus, the discovery of natural α-glucosidase inhibitors with fewer side effects is of significant importance for the effective and safe management of DM.

Astragali Radix (AR) is derived from the root of *Astragalus membranaceus* (Fisch.) Bge. var. *mongholicus* (Bge.) Hsiao or *A. membranaceus* (Fisch.) Bge. [9], a medicine and food homology substance widely used in clinical practice for the treatment of DM in China [10,11]. It is also commonly incorporated into functional foods or dietary supplements for the adjunctive management of DM worldwide [12,13]. Among its constituents, flavonoids are considered the primary active components responsible for its hypoglycemic effects [14]. To date, 20 flavonoids related to AR have been reported to exhibit α-glucosidase inhibitory activity, while 13 have been reported to possess hypoglycemic effects. Although some flavonoids exhibit α-glucosidase inhibitory activity, their relatively weak effects limit their potential for practical application and development. For instance, the IC_50_ (the half maximal inhibitory concentration) of Aloeresin A is 11.94 mM, while aquilarixanthone has an IC_50_ of 678.14 μM [15]. Notably, emerging evidence suggests that flavonoids with α-glucosidase inhibitory properties may enhance the activity of other α-glucosidase inhibitors, potentially improving their efficacy [16]. For example, Han et al. reported synergistic effects between acarbose and the three active compounds, including myricetin, isoquercitrin, and kaempferol-3-*O*-rutinoside [17]. Additionally, the cooperative inhibition of α-glucosidase has been reported with the combination of total alkaloids and either catechin or quercetin [18]. Currently, no studies have explored the mixture effects of multiple flavonoid compounds.

In this study, we identified 16 compounds with α-glucosidase inhibitory activity from a total of 29 flavonoids related to AR, including 27 original constituents and two metabolites of daidzein formed in the gut. The inhibitory mechanisms of these compounds on α-glucosidase were examined using kinetic analysis, CD spectroscopy, SPR, and molecular docking. Moreover, our findings revealed that combining multiple flavonoid compounds at ineffective concentrations resulted in significant α-glucosidase inhibition. The hypoglycemic effects of the active compounds were further validated in a postprandial hyperglycemic mouse model. Overall, this study provides a theoretical foundation for the development of AR-related flavonoids as functional food ingredients or potential therapeutic agents for diabetes.

## 2. Results and Discussion

### 2.1. Inhibition Activity of Flavonoids on α-Glucosidase

**Figure 1:**
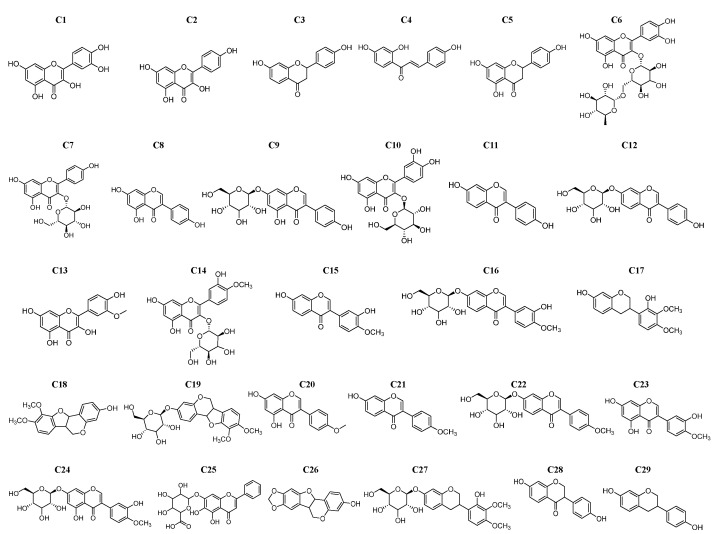
Structures of 29 AR-related flavonoid compounds (including 27 original constituents and 2 metabolites).

From 29 AR-related flavonoids, including 27 original constituents and 2 metabolites (**C28** and **C29**, the metabolites of daidzein in the gut), 16 compounds (**C1**–**C8**, **C10**, **C11**, **C14**, **C16**, **C17**, **C19**, **C28**, and **C29**) were screened out to have inhibitory effects on α-glucosidase (Figure 1, Table 1). The compounds were ranked by inhibitory strength as follows: **C1** > **C2** > **C8** > **C4** > **C10** > **C11** > **C29** > **C3** > **C7** > **C6** > **C5** > **C14** > **C17** > **C19** > **C28** > **C16**. The IC_50_ values of **C5**, **C11**, **C14,** and **C16** were consistent or nearly consistent with the previous report (Table 1). Seven compounds (**C1**, **C2**, **C4**, **C6**–**C8**, and **C10**), along with acarbose, exhibited IC_50_ values within the range reported in the literature (Table 1). Notably, **C3**, **C17**, **C19**, **C28,** and **C29** were reported for the first time as α-glucosidase inhibitors, with their IC_50_ values of 131.37 ± 7.65 μM, 160.77 ± 36.29 μM, 347.67 ± 32.36 μM, 354.26 ± 40.38 μM, and 412.00 ± 11.40 μM, respectively. Quercetin (**C1**) demonstrated the strongest inhibitory activity, with the lowest IC_50_ value (6.65 ± 0.43 μM), followed by kaempferol (**C2**, 38.79 ± 4.96 μM) and genistein (**C8**, 64.80 ± 0.27 μM). In contrast, calycosin-7-*O*-glucoside (**C16**) exhibited the weakest activity, with the highest IC_50_ value (563.40 ± 43.56 μM). Although the IC_50_ of these flavonoids was higher than that of acarbose in this study, they offer distinct advantages, including natural origin, low cost, and fewer side effects. Moreover, previous studies have suggested the potential of flavonoids as bioactive dietary compounds [19,20]. For instance, **C1** is abundant in onions, apples, broccoli, and berries, while **C5** is found in citrus fruits and peels [21]. Notably, **C28** and **C29,** as metabolites of daidzein, expand our understanding of how flavonoid metabolism may influence α-glucosidase inhibition. Taken together, these findings not only enrich the catalog of AR-related flavonoids with α-glucosidase inhibitory potential but also provide new leads for SAR studies.

### 2.2. Structure–Activity Relationship (SAR) Analysis

**Figure 2:**
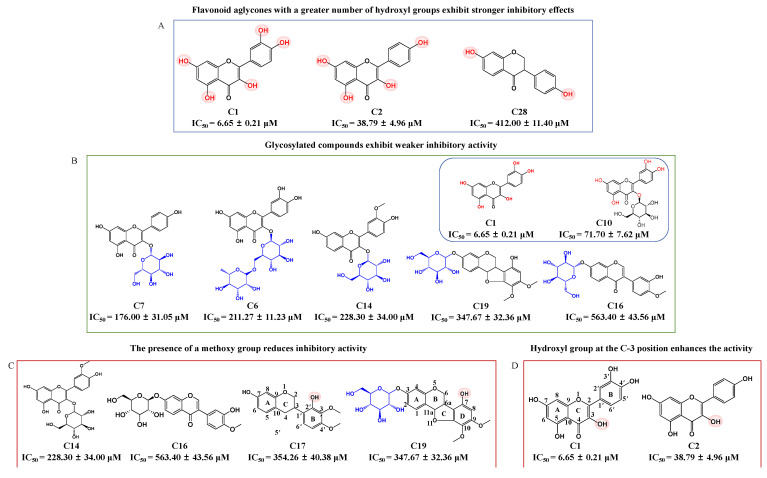
The effect of the structure of AR-related flavonoid compounds on α-glucosidase inhibitory activity. (**A**) Flavonoid aglycones with a greater number of hydroxyl groups exhibit stronger inhibitory effects; (**B**) Glycosylated compounds exhibit weaker inhibitory activity; (**C**) The presence of a methoxy group reduces inhibitory activity; (**D**) Hydroxyl group at the C-3 position enhances the activity.

In order to guide the structural optimization and synthesis of compounds, the SARs of 16 flavonoid compounds exhibiting α-glucosidase inhibitory activity were analyzed (Figure 2). This analysis confirms that both the number of hydroxyl groups and glycosylation influence inhibitory activity. Flavonoid aglycones with a greater number of hydroxyl groups, such as **C1** and **C2**, exhibited stronger inhibitory effects, whereas **C28**, which contains only two hydroxyl groups, displayed weaker inhibitory activity (Figure 2A). While hydroxylation of flavonoids improves their inhibitory activity, glycosylation tends to reduce it. For example, **C1** exhibited stronger inhibitory activity than its glycosylated derivative, **C10**. Similarly, glycosylated compounds like **C6**, **C7**, **C14**, **C16**, and **C19**, with IC_50_ values greater than 100 μM, displayed weaker inhibitory effects (Figure 2B). These results are consistent with previous reports [39]. Additionally, the presence of a methoxy group was found to reduce inhibitory activity, as observed in less active compounds such as **C14**, **C16**, **C17,** and **C19** (Figure 2C). Moreover, this study is the first to demonstrate that the presence of a hydroxyl group at the C-3 position of the C-ring enhances inhibitory activity, as observed with **C1** and **C2** (Figure 2D), the two most potent active compounds among the 16 inhibitors. For example, myricetin, which has a hydroxyl group at the C-3 position, exhibited the strongest inhibitory effect on α-glucosidase among eight flavonoid compounds [17]. Similarly, 6-geranyl-3,3′,5,5′,7-pentahydroxy-4′-methoxyflavane, containing the highest number of hydroxyl groups, including one at the C-3 position, was identified as the most potent α-glucosidase inhibitor among eight flavonoid compounds [40]. This discovery offers new perspectives for the development and synthesis of future compounds. In summary, the inhibitory activity of flavonoid compounds is closely associated with their chemical structures, particularly the number and position of hydroxyl groups, as well as the presence of glycosylation and methoxy groups. These findings offer important guidance for the rational design of more potent α-glucosidase inhibitors.

### 2.3. Kinetic Type of Inhibition on α-Glucosidase

**Figure 3:**
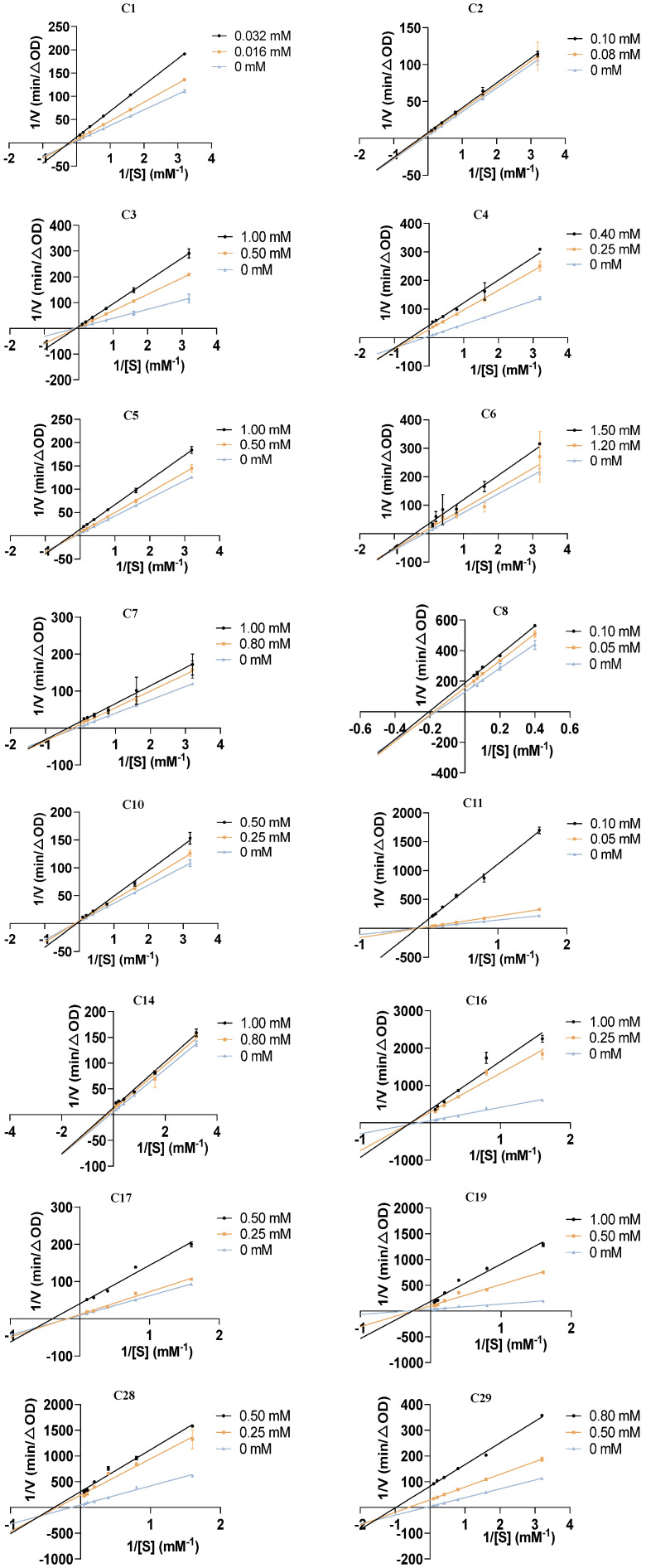
Lineweaver–Burk plots illustrating the inhibitory kinetics of 16 AR-related flavonoid compounds on α-glucosidase.

In order to further uncover the inhibitory mechanisms of the 16 flavonoids against α-glucosidase, Lineweaver–Burk plots were used to analyze their inhibitory type on α-glucosidase. As shown in Figure 3, for compounds **C1**, **C2**, **C4**, **C5**–**C8**, **C14**, **C16**, **C17**, **C28**, and **C29**, the double-reciprocal plots of reaction velocity (v) against varying concentrations of the substrate (pNPG) revealed that the data lines intersected in the third quadrant. Additionally, both the vertical intercept (1/V_max_) and horizontal intercept (1/K_m_) changed simultaneously, indicating a decrease in V_max_ and an increase in K_m_. These findings suggest that these 12 flavonoid compounds act as mixed-type inhibitors of α-glucosidase, including competitive and non-competitive inhibition, meaning they can bind to both the free enzyme and the enzyme–substrate complex. In contrast, compounds **C3**, **C11**, and **C19** showed a decrease in V_max_, while K_m_ remained constant as inhibitor concentration increased, with data lines intersecting on the negative direction of the horizontal axis. This pattern indicates that these three compounds function as non-competitive inhibitors of α-glucosidase, binding to the free enzyme. For **C10**, an increase in concentration resulted in a increase in the slope, while the vertical intercept remained unchanged. The data lines nearly intersected at a single point on the vertical axis, indicating that **C10** is a competitive inhibitor of α-glucosidase. These results further demonstrate that the 16 flavonoids are potent α-glucosidase inhibitors with different kinetic types. Notably, the inhibitory types of **C3**, **C16**, **C17**, **C19**, **C28**, and **C29** on α-glucosidase have not been previously reported, making this study the first to investigate their inhibitory mechanisms.

### 2.4. Circular Dichroism (CD) Spectra

**Figure 4:**
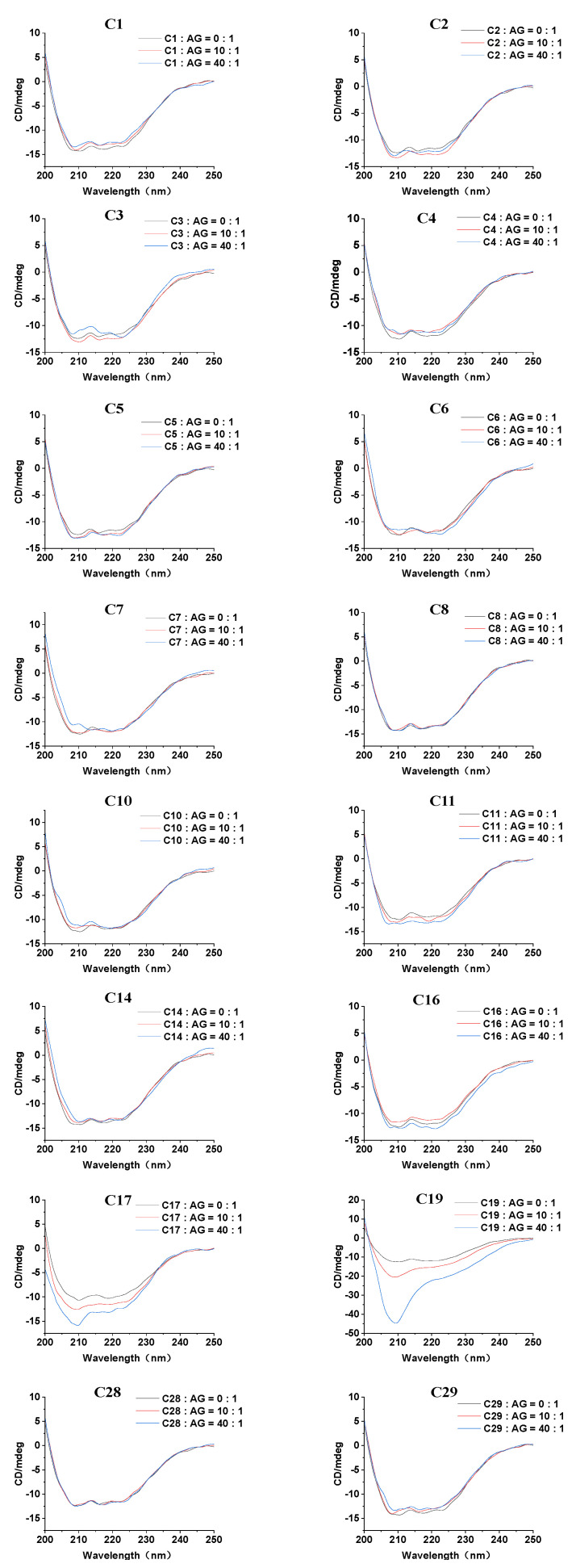
Effects of 16 AR-related flavonoid compounds on the CD spectra of α-glucosidase (AG).

To investigate the influence of the 16 flavonoids on the secondary structure of α-glucosidase, CD spectroscopy was employed as a sensitive and effective technology [41]. As shown in Figure 4, the free α-glucosidase exhibited two characteristic negative bands around 208 nm and 222 nm in the CD spectrum, which are characteristics of α-helix and are attributed to the π–π* and n–π* electron transitions, respectively [42]. Upon the addition of various concentrations of the 16 flavonoid inhibitors, changes in band intensity in the CD spectra were observed, indicating alterations in the enzyme’s conformation. Additionally, our study is the first to reveal the CD spectrum changes of α-glucosidase induced by **C3**, **C4**, **C11**, **C14**, **C17**, **C19**, **C28**, and **C29**. For the remaining compounds, our results are generally consistent with previous studies, which also reported flavonoid-induced conformational changes in α-glucosidase [17,36,43,44].

The secondary structure percentages of α-glucosidase were calculated using the BeStSel database (Table S1) [23,45]. For free α-glucosidase, the proportions of α-helix, β-sheet, β-turn, and random coil were 14.4%, 28.8%, 11.7%, and 45.2%, respectively, which is consistent with previous reports [27,46]. Specific structural changes were observed upon treatment with the 16 flavonoids. For instance, when **C14** and **C16** were incubated with α-glucosidase at a 10:1 molar ratio, the α-helix content decreased from 14.4% to 13.1% and 13.3%, while the β-sheet content increased from 28.8% to 33.7% and 29.5%, respectively. The results implied that the flavonoids disrupted the hydrogen-bonding network of α-glucosidase, leading to a more relaxed enzyme conformation that impairs its substrate-binding capacity and reduces catalytic activity [17]. In contrast, treatment with other set inhibitors, including **C1**, **C2**, **C3**, **C5**, **C10**, **C28**, and **C29**, caused simultaneous decreases in both α-helix and β-sheet content, whereas **C4** increased both α-helix and β-sheet content. These results further indicate that alterations in the secondary structure of α-glucosidase induced by these AR-related flavonoids can also affect enzyme binding and inhibit its activity.

### 2.5. Surface Plasmon Resonance (SPR) Analysis

**Figure 5:**
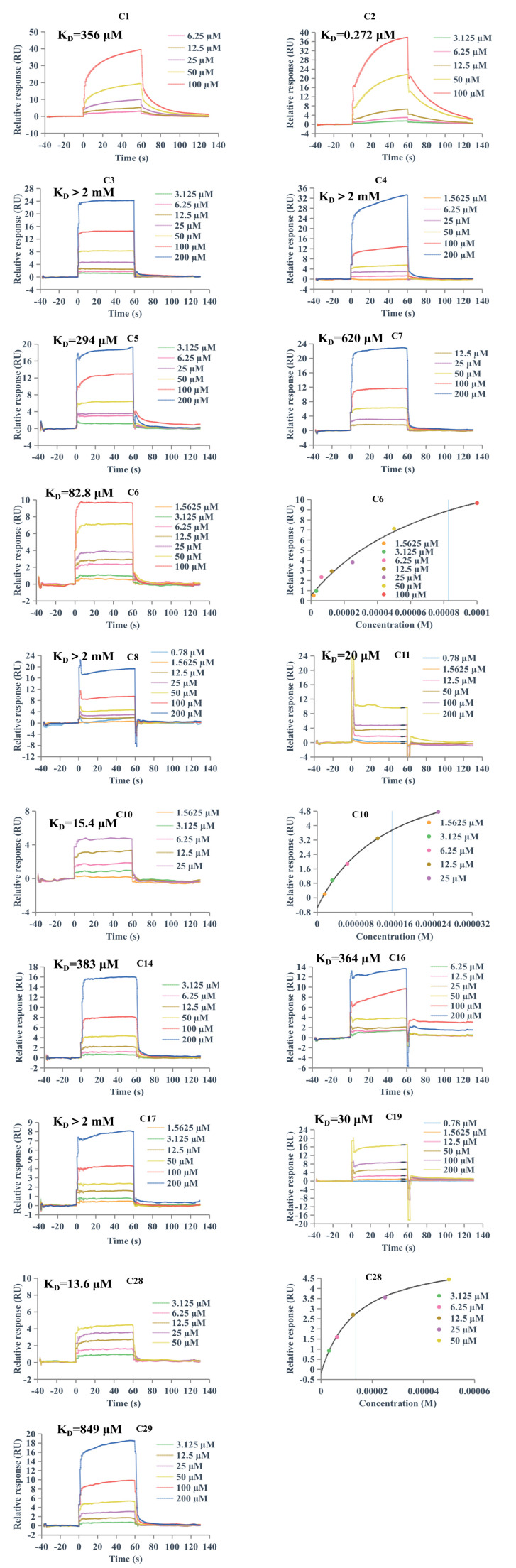
Binding kinetics and affinity of 16 AR-related flavonoids to α-glucosidase by SPR assay.

SPR spectroscopy is a powerful technique for analyzing biomolecular binding interactions [47]. In this study, we employed SPR assay to evaluate the direct binding affinity of 16 AR-related flavonoids to α-glucosidase for the first time. As shown in Figure 5, **C1**, **C2**, **C5**–**C7**, **C10**, **C11**, **C14**, **C16**, **C19**, **C28**, and **C29** exhibited concentration-dependent binding to α-glucosidase, with *K*_D_ values determined to be 356 μM, 0.272 μM, 294 μM, 82.8 μM, 620 μM, 15.4 μM, 20 μM, 383 μM, 364 μM, 30 μM, 13.6 μM, and 849 μM, respectively. The *K*_D_ values of **C6**, **C10**, and **C28** were calculated using the steady-state affinity model, while the remaining compounds were analyzed using the kinetic model. These results suggest that these 12 flavonoids possess the capacity to bind to α-glucosidase. In contrast, the remaining four compounds, **C3**, **C4**, **C8**, and **C17**, did not show binding to α-glucosidase, which may be attributed to the conformation of the protein in this assay, where α-glucosidase was present in a bound state rather than as a free protein.

### 2.6. Molecular Docking Analysis

**Figure 6:**
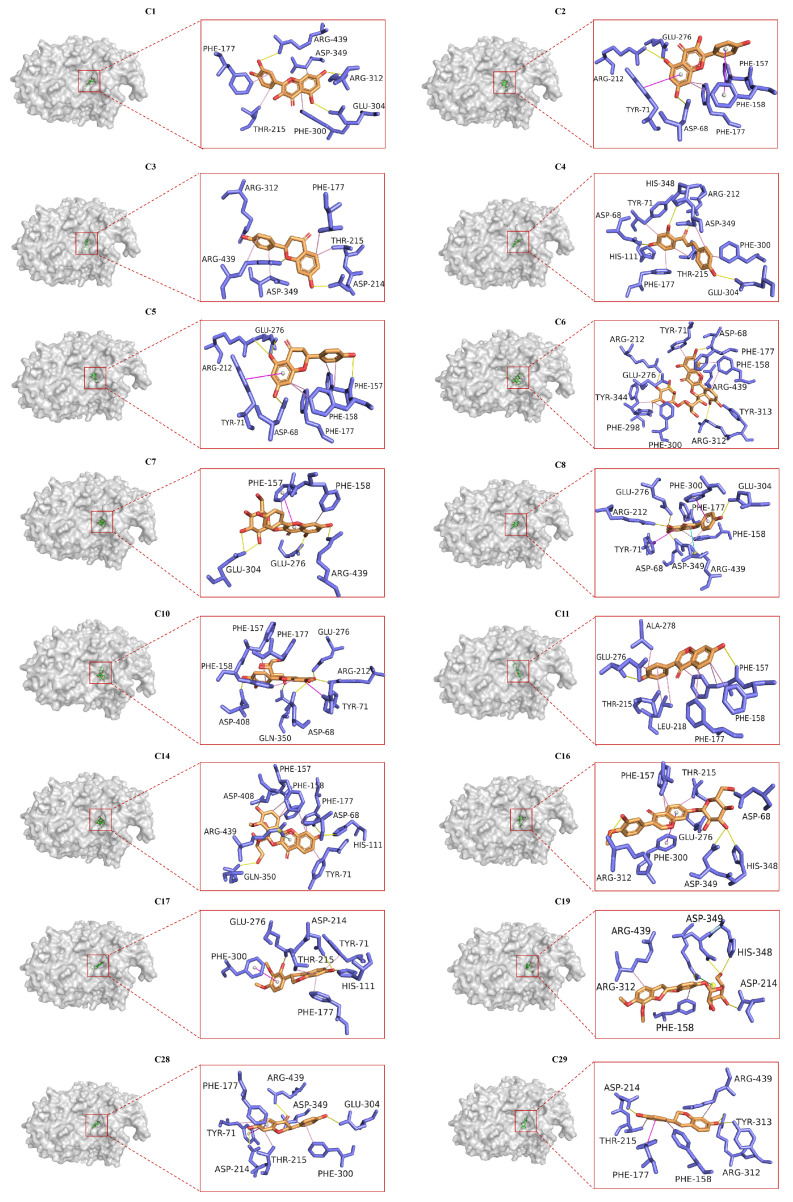
Molecular docking results of 16 AR-related flavonoid compounds within the active pocket of α-glucosidase. Yellow lines indicate hydrogen bonds, green lines represent salt bridges, pink lines indicate hydrophobic interactions, purple lines represent π–π stacking interactions, and blue lines represent the cation–π interactions.

Molecular docking was used to simulate the binding mode of the ligands and receptors based on the “lock” and “key” principle [41]. In order to predict the interactions between the 16 AR-related flavonoids, acarbose, and α-glucosidase, docking studies were carried out. The most populated conformer, with the lowest free energy and best docking score, was selected for further analysis. The results showed that the 16 flavonoids (Figure 6) and acarbose (Figure S1) were all embedded within the active pocket of the α-glucosidase, forming tight interactions with surrounding amino acid residues through hydrogen bonding, hydrophobic interactions, π–π stacking, salt bridges, and cation–π interactions, thereby influencing the enzyme’s activity. Additionally, this study is the first to report the molecular docking analysis of **C3**, **C14**, **C17**, **C19**, **C28**, and **C29** with α-glucosidase, revealing the specific amino acid residues and binding modes involved in their interactions. The binding energies and binding sites of the 16 flavonoids are detailed in Table S2. The docking score of acarbose was −6.63 kcal·mol^−1^, consistent with the previously reported value of −6.32 kcal·mol^−1^ [48], suggesting a strong binding affinity. Generally, lower binding energies indicate a stronger ligand affinity for the catalytic center [42]. Notably, **C1**, **C3**–**C5**, **C8**, **C11**, **C14**, **C16**, **C17**, **C28**, and **C29** demonstrated more stable complexes with α-glucosidase than acarbose, as indicated by their lower docking scores (Table S2). In contrast, **C6**, **C7**, **C10**, and **C19** exhibited slightly weaker binding affinities than acarbose, likely due to the presence of glycosyl groups and their larger molecular weight, which may hinder efficient insertion into the active site. Although **C2** has a smaller molecular weight, it also showed weaker binding than acarbose, possibly due to fewer hydrogen bonds formed with α-glucosidase. Further analysis identified Asp68, Arg212, Asp214, Glu276, and Asp349 as key amino acid residues crucial for the inhibitory effects of these compounds on α-glucosidase. Most compounds bound to these residues, suggesting their critical role in the enzyme’s inhibition.

### 2.7. Inhibitory Effects of Combinations of Flavonoid Compounds on α-Glucosidase

**Figure 7:**
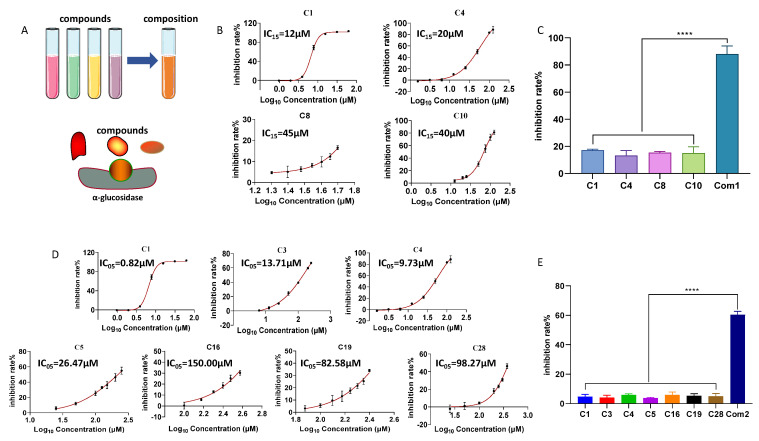
Inhibitory effects of flavonoid compound combinations on α-glucosidase. (**A**) Schematic representation of the combination of flavonoid compounds with α-glucosidase. (**B**) Dose–response curves for **C1**, **C4**, **C8**, and **C10**. (**C**) Inhibition by the combination of **C1**, **C4**, **C8**, and **C10** at IC_15_ concentrations. (**D**) Dose–response curves for **C1**, **C3**, **C4**, **C5**, **C16**, **C19**, and **C28**. (**E**) Inhibition by the combination of **C1**, **C3**, **C4**, **C5**, **C16**, **C19**, and **C28** at IC_05_ concentrations. Data are presented as mean ± SD. **** *p* < 0.0001, compared with individual compounds, as determined by ANOVA.

The results of this study, along with findings from the literature [49], suggest that certain flavonoid compounds related to AR, such as **C16**, **C28**, and calycosin-7-*O*-glucoside-6″-*O*-malonate [49], exhibit weak inhibitory activity against α-glucosidase. This limited activity may hinder their potential use in the development of antidiabetic drugs or functional foods. However, based on the “additive effect theory” hypothesis [50], which posits that combining multiple compounds at low or ineffective concentrations—regardless of their individual inhibitory strength—can produce a significant pharmacological effect. We first combined four stronger inhibitors—**C1**, **C4**, **C8**, and **C10**—at ineffective concentrations (IC_15_, the concentration corresponding to a 15% inhibition rate, calculated from the dose–response curve, Figure 7B); the individual inhibition rates of these compounds against α-glucosidase were 17.16 ± 0.75%, 13.32 ± 3.55%, 15.37 ± 0.83%, and 14.93 ± 4.73%, respectively (Figure 7C). In contrast, their mixture (Composition 1, Com1) exhibited a significantly enhanced inhibition rate of 88.04 ± 5.98% (Figure 7C). The combination index (CI) for Com1 was 0.82, indicating a significant synergistic effect. Additionally, we combined seven compounds, with stronger and weaker activities—**C1**, **C3**, **C4**, **C5**, **C16**, **C19**, and **C28**—at IC_05_ concentrations (calculated from the dose–response curve, Figure 7D). The individual inhibition rates for these compounds were as follows: **C1** (4.70 ± 1.66%), **C3** (4.18 ± 1.50%), **C4** (6.05 ± 0.63%), **C5** (3.88 ± 0.40%), **C16** (5.95 ± 1.95%), **C19** (5.35 ± 1.42%), and **C28** (5.05 ± 1.87%) (Figure 7E). In comparison, their mixture (Composition 2, Com2) achieved an inhibition rate of 60.38 ± 2.33%, with a CI value of 1.00, indicating an additive effect. These findings demonstrate that, regardless of the individual inhibitory activity of the flavonoid compounds, combining multiple flavonoids at ineffective concentrations can significantly enhance their α-glucosidase inhibitory effects. This insight provides a promising approach for developing highly effective, low-toxicity antidiabetic drugs and functional foods derived from AR-related flavonoids.

### 2.8. Effects of 16 Flavonoids on Postprandial Hyperglycemia in Normal Mice

**Figure 8:**
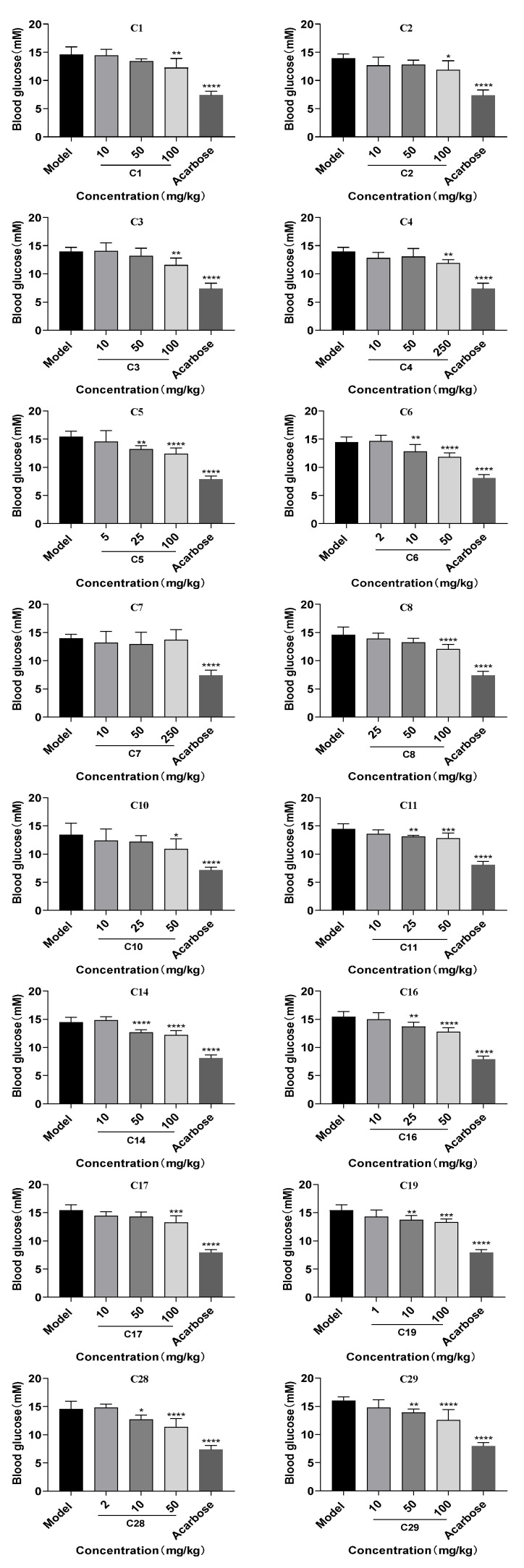
Effects of 16 AR-related flavonoid compounds on postprandial blood glucose levels in normal mice following oral sucrose administration. Data are presented as mean ± SD (n = 8). * *p* < 0.05, ** *p* < 0.01, *** *p* < 0.001, and **** *p* < 0.0001, compared with the model group by ANOVA.

While the α-glucosidase inhibitory activity of 16 flavonoids was confirmed in vitro, their potential to reduce postprandial hyperglycemia in vivo required further investigation. As shown in Figure 8, 15 out of the 16 flavonoids (**C1**–**C6**, **C8**, **C10**, **C11**, **C14**, **C16**, **C17**, **C19**, **C28**, and **C29**) significantly reduced postprandial blood glucose levels at 30 min after the sucrose load compared to the model group, and the positive control group (acarbose) reduced blood glucose levels by 44.13% to 50.02%. Notably, **C3**, **C16**, **C17**, **C19**, and **C28** were identified for the first time in this study as compounds capable of lowering blood glucose levels. Their effects on postprandial blood glucose at 0, 30, 60, 90, and 120 min are shown in Figure S2. Among the 15 effective flavonoids, 8 compounds (**C5**, **C6**, **C11**, **C14**, **C16**, **C19**, **C28**, and **C29**) exhibited a glucose-lowering rate of 9.33–21.87% at medium and high doses, showing a dose-dependent effect. In contrast, the remaining seven compounds exhibited glucose-lowering effects only at the highest dose; namely, **C1**–**C3**, **C8**, and **C17** reduced blood glucose by 15.87%, 14.80%, 16.95%, 17.24%, and 14.25% only at 100 mg/kg, respectively; **C4** reduced blood glucose levels by 14.62% at 250 mg/kg, and **C10** lowered glucose by 18.82% at 50 mg/kg. The blood glucose values of the 15 compound groups at different concentrations are shown in Table S3.

Combined with the results of α-glucosidase inhibition assays, these 15 compounds are speculated to inhibit α-glucosidase activity, thereby slowing the hydrolysis of sucrose into glucose and delaying intestinal glucose absorption, ultimately exerting a hypoglycemic effect. Among these, **C10** exhibited strong α-glucosidase inhibitory activity in vitro, with an IC_50_ value of 71.10 μM. It also demonstrated a blood glucose reduction of 18.82% in vivo, second only to **C29**, demonstrating greater efficacy than other flavonoid inhibitors reported in the literature. For example, Tian et al. [51] found that quercetagetin-7-*O*-glucoside, which shares structural similarity with **C10**, exhibited significant hypoglycemic effects, reducing blood glucose by 16.7% at a dose of 50 mg/kg, which was lower than **C10** at the same dose. Similarly, Xu et al. [52] reported that another flavonoid, pelargonidin-3-*O*-rutinoside, showed significant hypoglycemic effects, reducing blood glucose by 18.5% at 50 mg/kg, also lower than **C10** at the same dose. Taken together, these findings suggest that **C10** possesses anti-hyperglycemic potential, indicating its promising application as an anti-diabetic compound. However, **C7** showed no glucose-lowering effect at doses of 10, 50, or 250 mg/kg. This lack of effect for **C7** could be attributed to its metabolism, which could result in insufficient in vivo concentrations to exert a hypoglycemic effect. Additionally, the complexity of the in vivo environment, including factors such as other enzymes, gut microbiota, and metabolic pathways, is known to influence drug efficacy [53,54].

## 3. Materials and Methods

### 3.1. Chemicals and Materials

The tested compounds included quercetin (**C1**, Lot No. PS010462), kaempferol (**C2**, Lot No. PS011676), liquiritigenin (**C3**, Lot No. PS020823), isoliquiritigenin (**C4**, Lot No. PS001032), naringenin (**C5**, Lot No. PS010691), rutin (**C6**, Lot No. PS012206), astragalin (**C7**, Lot No. PS011379), genistein (**C8**, Lot No. PS012133), genistin (**C9**, Lot No. PS000781), isoquercitrin (**C10**, Lot No. PS011802), daidzein (**C11**, Lot No. PS000251), daidzin (**C12**, Lot No. PS000250), isorhamnetin (**C13**, Lot No. PS011340), isorhamnetin-3-*O*-glucoside (**C14**, Lot No. PS011411), calycosin (**C15**, Lot No. PS010251), calycosin-7-*O*-glucoside (**C16**, Lot No. PS010251), isomucronulatol (**C17**, Lot No. PS220427-09), astrapterocarpan (**C18**, Lot No. PS011553), astrapterocarpan-3-*O*-glucoside (**C19**, Lot No. PS020771), biochanin A (**C20**, Lot No. PS001112), formononetin (**C21**, Lot No. PS000674), ononin (**C22**, Lot No. PS000671), pratensein (**C23**, Lot No. PS010890), pratensein-7-*O*-glucoside (**C24**, Lot No. PS1638), baicalin (**C25**, Lot No. PS011251), and maackiain (**C26**, Lot No. PS012519), all of which were purchased from PUSH Bio-Technology (Chengdu, China); isomucronulatol-7-*O*-glucoside (**C27**, Lot No. PS220112-01) was purchased from Chengdu Pufeide Biotech Co., Ltd.; dihydrodaidzein (**C28**, Lot No. 022041088) was purchased from Mreda (Beijing, China), and equol (**C29**, Lot No. P1748942) was purchased from Adamas (Shanghai, China). All of these flavonoid compounds had a purity of ≥98% as determined by peak area normalization using Q-TOF detection. Saccharomyces cerevisiae α-glucosidase (33 U/mg, Lot No. M23HS178879), acarbose (Lot No. 56180-94-0), and p-nitrophenyl-α-D-glucopyranoside (pNPG, Lot No. J29GS156009) were obtained from Shanghai Yuanye Biotechnology Co., Shanghai, China. Dimethyl sulfoxide (DMSO, Lot No. SHBM5161) was purchased from Sigma-Aldrich (St. Louis, MO, USA).

### 3.2. α-Glucosidase Inhibition Assay

The α-glucosidase inhibitory effects of flavonoids were evaluated as in a previous report with slight modifications (including changes in the concentrations of the enzyme, substrate, and Na_2_CO_3_) [55]. Briefly, 50 μL of phosphate-buffered saline (PBS, 0.1 M, pH 6.8), the test compound, and α-glucosidase (0.2 U/mL) were preincubated in a 96-well plate at 37 °C for 10 min. The reaction was then initiated by adding 50 μL of 5 mM pNPG as the substrate. Subsequently, the 96-well plate was incubated for an additional 30 min at 37 °C, then 50 μL of 0.2 M Na_2_CO_3_ was added to terminate the reaction. Absorbance was measured at under 405 nm. Acarbose was used as a positive control, and all tested compounds were dissolved in 5% DMSO. The α-glucosidase inhibition rate was calculated using the following Formula (1) [56]:

(1) $$\mathrm{inhibition}\;\mathrm{rate}(\%)=1-\frac{A_{s}-A_{sc}}{A_{b}-A_{bc}}\times 100\%$$

where *A_s_*, *A_sc_*, *A_b_*, and *A_bc_* represent the absorbance of the sample group (containing the PBS buffer, enzyme, compounds, pNPG, and Na_2_CO_3_ solution), the sample control group without pNPG, the blank group without the compounds, and the blank control group without the compounds and pNPG, respectively.

### 3.3. Inhibitory Kinetic Analysis

The type of inhibition was determined by using the kinetics curve and the Lineweaver–Burk plot [56]. Various concentrations of the compounds were tested: **C1** (0, 0.016, and 0.032 mM); **C2** (0, 0.08, and 0.1 mM); **C3**, **C5**, and **C19** (all at 0, 0.50, and 1.00 mM); **C4** (0, 0.25, and 0.40 mM); **C6** (0, 1.2, and 1.5 mM); **C7** and **C14** (both at 0, 0.80, and 1.00 mM); **C8** and **C11** (both at 0, 0.05, and 0.10 mM); **C10**, **C17**, and **C28** (all at 0, 0.25, and 0.50 mM); **C16** (0, 0.25, and 1.00 mM); **C29** (0, 0.50, and 0.80 mM). These compounds were incubated with α-glucosidase at 37 °C for 30 min. After incubation, various concentrations of pNPG (0.31–20 mM) were added, and absorbance was measured at 405 nm. The Lineweaver–Burk plot was obtained by plotting 1/[S] on the *x*-axis and 1/v on the *y*-axis, where [S] represents substrate concentration and v represents the reaction velocity.

### 3.4. CD Spectroscopy

CD spectra in the far-UV region (190–250 nm) were scanned by using a CD spectrometer (JASCO-1.5 Tokyo, Japan). The α-glucosidase was mixed at a concentration of 4 μM, and the molar ratios of the 16 flavonoid compounds (**C1**–**C8**, **C10**, **C11**, **C14**, **C16**, **C17**, **C19**, **C28**, and **C29**) to α-glucosidase were set to 0:1, 10:1, and 40:1, respectively. The slit width was set to 1 nm, and the baseline was calibrated with PBS (phosphate buffered saline). CD spectra were averaged over three scans under a constant nitrogen flow, with the buffer signal subtracted [57]. The results were analyzed using the BeStSel database (https://bestsel.elte.hu/index.php, accessed on 16 May 2025).

### 3.5. SPR Experiments

SPR analysis was performed using the Biacore 8K system (GE Healthcare, Uppsala, Sweden). α-Glucosidase (0.05 mg/mL) was fixed on the CM5 sensor chip by an amine coupling kit. Subsequently, various concentrations of the 16 flavonoids dissolved in 5% DMSO were continuously injected onto the surface of α-glucosidase coated surface. After each sample injection, the sensor chip surface was washed and regenerated with running buffer (PBS) at a flow rate of 30 µL/min. According to the 1:1 Langmuir model, the equilibrium dissociation constant (*K*_D_) was calculated by the Biacore evaluation software. For sensorgrams without significant kinetic features, the *K*_D_ values were calculated using the steady-state analysis.

### 3.6. Molecular Docking

The protein structure was modeled using the Swiss-Model server (https://swissmodel.expasy.org/interactive, accessed on 15 April 2023) with homology modeling based on the X-ray structure of isomaltase from Saccharomyces cerevisiae (PDB ID: 3AJ7) [58], which shares 72.4% sequence identity with the target protein. The final model was optimized using PROCHECK (https://saves.mbi.ucla.edu/, accessed on 17 May 2025). The ligand-binding site was identified to include the following amino acid residues: Asp69, His112, Arg213, Asp215, Glu277, His351, Asp352, and Arg442 [59].

All water molecules were removed, and hydrogen atoms were added to the protein. The 3D structures of the 16 flavonoids were constructed using ChemBio3D Ultra 14.0. To prepare for docking with the AutoDockTools-1.5.6, both the ligand and the enzyme structures were converted to pdbqt file format using OpenBabel-3.1.1. After that, the grid size was set to 60 × 50 × 64 Å with a grid spacing of 0.375 Å. The center of the grid box was positioned at the Cartesian coordinates x = 21.5, y = −5.687, and z = 23.556. Docking calculations were performed using the Lamarckian genetic algorithm (LGA), with 100 runs. The binding model with the lowest energy was selected for further analysis to explore the action mechanisms of the 16 flavonoids on α-glucosidase.

### 3.7. Mixture Effects

The inhibitory activity of the composition was evaluated using the same method described in Section 3.2. Flavonoids related to AR were combined at ineffective concentrations to evaluate their inhibitory effect on α-glucosidase. First, four stronger inhibitors—**C2**, **C4**, **C8**, and **C10**—were combined at IC_15_ to form Composition 1. The IC_15_ values for these compounds, determined from the dose–response curve and calculated using the Formula (2), were 12 μM, 20 μM, 45 μM, and 40 μM, respectively, resulting in a final concentration of 117 μM for Composition 1. Next, 7 compounds, regardless of their individual strengths (**C1**, **C3**, **C4**, **C5**, **C16**, **C19**, and **C28**), were combined at IC_05_ to form Composition 2. The IC_05_ concentrations of these compounds were 0.82 μM, 13.71 μM, 9.73 μM, 26.47 μM, 150.00 μM, 82.58 μM, and 98.27 μM, respectively, resulting in a final concentration of 381.58 μM for Composition 2. The combination index (CI, Formula (3)) was subsequently used to assess the interactions between the compounds [60].

(2) Y=Bottom+Top−Bottom1+10^((LogIC50−X)×HillSlope)

In Formula (2), Bottom represents the baseline response, Top denotes the maximum response, LogIC_50_ is the logarithmic transformation of IC_50_, and HillSlope refers to the slope of the dose–response curve.

(3) $$\mathrm{CI}=\sum_{j=1}^{n}\frac{{\left(D\right)}_{j}}{{\left(D_{x}\right)}_{j}}$$

In Formula (3), (D)_j_ represents the concentration of component j in the mixture needed to achieve an effect of x, while (D_x_)_j_ denotes the concentration of component j required to produce the same effect when acting alone. Here, x refers to the inhibition rate produced by the composition, and n indicates the number of individual compounds in the composition.

### 3.8. Experimental Animals

Male C57BL/6J mice of SPF grade, aged 6–8 weeks and weighing 18–22 g, were obtained from the Experimental Animal Science Department of Peking University. The mice were housed in a controlled environment with a 12:12 light/dark cycle and a constant temperature. They were provided with standard pellet feed and given free access to drinking water. To ensure acclimatization, the mice were allowed to adjust to the diet and housing conditions for one week prior to the experiment. All animal experiments were performed in compliance with the relevant laws and institutional guidelines for the care and use of laboratory animals in China [61]. The experiments were approved by the Laboratory Animal Management and Ethics Committee of Peking University (LA2021443). Additionally, the certification number for the laboratory animal personnel is 1120111300011.

### 3.9. Blood Analysis

The 16 flavonoids were subsequently evaluated for their potential antihyperglycemic effects by using the oral sucrose test, which is commonly used to evaluate intestinal α-glucosidase inhibition in vivo [62]. Normal C57BL/6J mice were randomly divided into model groups and the 16 flavonoid compound groups (n = 8 per group), respectively. The flavonoids at different concentrations were suspended in 0.5% sodium carboxymethyl cellulose (CMC-Na). After fasting for 16 h, the mice were orally administered one of the following compounds via a stomach tube: acarbose at 20 mg/kg; **C1**–**C3**, **C14**, **C17**, and **C29** (all at 10, 50, and 100 mg/kg); **C4** and **C7** (both at 10, 50, and 250 mg/kg); **C5** (5, 25, and 100 mg/kg); **C6** and **C28** (both at 2, 10, and 50 mg/kg); **C8** (25, 50, and 100 mg/kg); **C10**, **C11**, and **C16** (all at 10, 25, and 50 mg/kg); and **C19** (1, 10, and 100 mg/kg). Fifteen minutes after drug administration, the mice were given a sucrose solution (4 g/kg). Blood samples were collected from the tail vein at 30 min after sucrose loading, and blood glucose levels were measured using an Accu-Chek Performa glucometer (Roche Diagnostics, SN: 79508621393, Shanghai, China). The rate of blood glucose reduction was calculated using the following Formula (4) [63]:

(4) $$\mathrm{The}\;\mathrm{rate}\;\mathrm{of}\;\mathrm{blood}\;\mathrm{glucose}\;\mathrm{reduction}\%=\frac{\mathrm{the}\;\mathrm{model}\;\mathrm{blood}\;\mathrm{glucose}-\mathrm{the}\;\mathrm{sample}\;\mathrm{blood}\;\mathrm{glucose}}{\mathrm{the}\;\mathrm{model}\;\mathrm{blood}\;\mathrm{glucose}}\times 100\%$$

### 3.10. Statistical Analysis

All data are expressed as the mean ± standard deviation (SD) based on three independent experiments. Statistical analyses were performed using GraphPad Prism 8.3 software (San Diego, CA, USA). Significances were analyzed using one-way analysis of variance (ANOVA), and *p* < 0.05 was considered to be statistically significant.

## 4. Conclusions

In this study, we screened 16 α-glucosidase inhibitors from a total of 29 AR-related flavonoids, including 14 original constituents and 2 metabolites (**C28** and **C29**). Among them, **C3**, **C17**, **C19**, **C28**, and **C29** are reported here for the first time as novel α-glucosidase inhibitors. The inhibitory mechanisms and SAR of these 16 active compounds were further investigated. The results revealed that the presence of hydroxyl groups, particularly at the C-3 position, greatly enhanced α-glucosidase inhibition. In contrast, the presence of glycosylation or a methoxy group reduced inhibitory activity. This study is the first to demonstrate the inhibitory types of **C3**, **C16**, **C17**, **C19**, **C28**, and **C29** against α-glucosidase. Additionally, the effects of **C4**, **C11**, **C14**, **C17**, **C19**, **C28**, and **C29** on the secondary structure of α-glucosidase were identified for the first time. The binding affinities of these 16 flavonoids for α-glucosidase were investigated using SPR, marking the first such analysis for these compounds. Molecular docking studies also provided novel insights into the binding sites of **C3**, **C14**, **C17**, **C19**, **C28**, and **C29** on α-glucosidase for the first time. These active compounds exhibited distinct inhibitory mechanisms, ultimately reducing α-glucosidase activity. Moreover, while some flavonoids demonstrated weak inhibitory activity when tested individually, combining multiple flavonoids at ineffective concentrations significantly enhanced their α-glucosidase inhibitory effects. In vivo studies further demonstrated that 15 of the 16 flavonoids reduced postprandial blood glucose levels in normal mice, with **C3**, **C16**, **C17**, **C19**, and **C28** reported for the first time. Collectively, these findings provide valuable insights for the future development of functional foods and therapeutic agents aimed at managing postprandial hyperglycemia.

## Figures and Tables

**Table 1:** α-Glucosidase inhibitory activity of 16 AR-related flavonoid compounds.

No.	Name	IC_50_ (μM)	IC_50_ in References (μM)
**C1**	quercetin	6.65 ± 0.21	3.3 [22], 17 [23], 230.3 [24]
**C2**	kaempferol	38.79 ± 4.96	18.6 [25], 420.5 [24], 1.22 × 10^3^ [26]
**C3**	liquiritigenin	160.77 ± 36.29	-
**C4**	isoliquiritigenin	69.11 ± 10.66	36.29 [27], 1.81 × 10^3^ [28]
**C5**	naringenin	225.83 ± 36.14	36.84 [29], 96.8 [25], 174 [30]
**C6**	rutin	211.27 ± 11.23	0.10 [25], 196 [25], 841.92 [31]
**C7**	astragalin	176.00 ± 31.05	114.6 [31], 519.21 [32]
**C8**	genistein	64.80 ± 24.24	50 [33], 70 [28], 150 [34]
**C10**	isoquercitrin	71.70 ± 7.62	47.40 [29], 185 [23]
**C11**	daidzein	83.66 ± 20.19	150 [28]
**C14**	isorhamnetin-3-*O*-glucoside	228.30 ± 34.00	275.4 [35]
**C16**	calycosin-7-*O*-glucoside	563.40 ± 43.56	174.04 [36]
**C17**	isomucronulatol	354.26 ± 40.38	-
**C19**	astrapterocarpan-3-*O*-glucoside	347.67 ± 32.36	-
**C28**	dihydrodaidzein	412.00 ± 11.40	-
**C29**	equol	131.37 ± 7.65	-
	acarbose	10.91 ± 0.36 nM	8 × 10^−3^ [37], 15.49 × 10^−3^ [26], 68.20 [38], 1.1 × 10^3^ [27]

“-” indicates that no studies have reported on this yet.

## Data Availability

The data presented in this study are available on request from the corresponding author. The data are not publicly available due to privacy.

## Supplementary Materials

The following supporting information can be downloaded at https://www.mdpi.com/article/10.3390/ph18050744/s1, Table S1: Content changes of α-glucosidase secondary structure under various concentrations of 16 AR-related flavonoids; Table S2: Binding energies and binding sites of 16 AR-related flavonoid compounds on α-glucosidase; Table S3: Effects of 15 AR-related flavonoid compounds on postprandial blood glucose levels in normal mice following oral sucrose administration; Figure S1: Molecular docking results of acarbose within the active pocket of α-glucosidase. Yellow lines indicate hydrogen bonds, green lines represent salt bridges, and pink lines indicate hydrophobic interactions; Figure S2: The effect of five newly identified flavonoids on postprandial blood glucose in mice at different time points. Significantly different from the control value, ** *p* < 0.05, ** *p* < 0.01, *** *p* < 0.001, **** *p* < 0.0001.

## Abbreviations

The following abbreviations are used in this manuscript:

AR	Astragali Radix
SAR	structure–activity relationship
CD	circular dichroism
SPR	surface plasmon resonance
DM	diabetes mellitus
IDF	International Diabetes Federation
pNPG	p-nitrophenyl-α-D-glucopyranoside
DMSO	dimethyl sulfoxide
PBS	phosphate-buffered saline
KD	dissociation constant
OSucTT	oral sucrose tolerance test
CMC-Na	sodium carboxymethyl cellulose
CI	combination index
PBS	phosphate buffered saline
