# Supplementary material for: α-Glucosidase Inhibition Mechanism and Anti-Hyperglycemic Effects of Flavonoids from Astragali Radix and Their Mixture Effects
Source: Pharmaceuticals (Basel). 2025 May 18;18(5):744. doi: 10.3390/ph18050744 (PMC12114633; doi:10.3390/ph18050744)
Supplement: Supplementary file 1 [file pharmaceuticals-18-00744-s001.zip › pharmaceuticals-3635111-supplementary.pdf]

Supporting Information

α-Glucosidase Inhibition Mechanism and Anti-Hyperglycemic Effects of Flavonoids from Astragali Radix and Their Mixture Effects

Xing Han [1], Pengpu Wang [1,2], Jing Zhang [1,3], Yang Lv [1], Zhigao Zhao [1,4], Fengxian Zhang [1,5], Mingying Shang [1], Guangxue Liu [1], Xuan Wang [6], Shaoqing Cai [1,*] and Feng Xu [1,7*]

[1] State Key Laboratory of Natural and Biomimetic Drugs, School of Pharmaceutical Sciences, Peking University, No. 38 Xueyuan Road, Beijing 100191, China; 2011110096@stu.pku.edu.cn (X.H.); 20240025@immu.edu.cn (P.-P.W.); 20241083@zcmu.edu.cn (J.Z.); victoryjack@pku.edu.cn (Y.L.); zhigaozhao@my.swjtu.edu.cn (Z.-G.Z.); 20210931204@bucm.edu.cn (F.-X.Z.); myshang@bjmu.edu.cn (M.-Y.S.); guangxl@bjmu.edu.cn (G.-X.L.);

[2] School of Public Health, Inner Mongolia Medical University, Hohhot 010110, China

[3] School of Pharmaceutical Sciences, Zhejiang Chinese Medical University, Hangzhou 310053, China

[4] College of Life Science and Engineering, Southwest Jiaotong University, Chengdu 610031, China

[5] School of Life Sciences, Beijing University of Chinese Medicine, Beijing 102488, China

[6] Department of Chemical Biology, School of Pharmaceutical Sciences, Peking University, No. 38 Xueyuan Road, Beijing 100191, China; xuanwang6818@bjmu.edu.cn (X.W.)

[7] Key Laboratory of State Administration of Traditional Chinese Medicine (TCM) for Compatibility Toxicology, Beijing, 100191, China

[*] Correspondence: sqcai@bjmu.edu.cn (S.-Q.C.); xufeng76@bjmu.edu.cn (F.X.); Tel.: +86-10-82801693 (S.-Q.C.); +86-10-82802534 (F.X.)

1. Table S1 Content changes of α-glucosidase secondary structure under various concentrations of 16 AR-related flavonoids

2. Table S2 Binding energies and binding sites of 16 AR-related flavonoid compounds on α-glucosidase

3. Table S3 Effects of 15 AR-related flavonoid compounds on postprandial blood glucose levels in normal mice following oral sucrose administration

4. Figure S1 Molecular docking results of acarbose within the active pocket of α-glucosidase. Yellow lines indicate hydrogen bonds, green lines represent salt bridges, and pink lines indicate hydrophobic interactions.

5. Figure S2 The effect of five newly identified flavonoids on postprandial blood glucose in mice at different time points. Significantly different from the control value, $^{**}P< 0.05$, $^{**}P< 0.01$, $^{***}P< 0.001$, $^{****}P< 0.0001$

Table S1 Content changes of α-glucosidase secondary structure under various concentrations of 16 AR-related flavonoids

NO.	Ratio*	α-helix (%)	β-sheet (%)	β-turn (%)	random coil (%)
α-glucosidase	0:1	14.4	28.8	11.7	45.2
**C1**	10:1	14.0	23.6	13.1	49.1
	40:1	11.1	27.9	12.5	48.5
**C2**	10:1	14.1	25.3	12.5	48.2
	40:1	9.70	23.9	13.3	53.1
**C3**	10:1	11.7	18.0	14.2	56.1
	40:1	8.00	16.6	16.2	59.2
**C4**	10:1	15.0	31.0	11.7	42.3
	40:1	14.1	24.1	12.1	49.7
**C5**	10:1	13.2	20.6	12.8	53.3
	40:1	15.3	25.3	13	46.5
**C6**	10:1	15.5	26.9	12.1	45.5
	40:1	9.30	14.3	17.6	58.8
**C7**	10:1	15.3	26.3	11.1	47.4
	40:1	9.00	23.1	14.0	53.9
**C8**	10:1	14.7	23.6	11.6	50.2
	40:1	16.1	16.9	13.0	54.1
**C10**	10:1	12.4	24.0	14.2	49.3
	40:1	12.5	16.6	13.1	57.8
**C11**	10:1	17.7	28.8	12.7	40.8
	40:1	14.6	25.5	14.3	45.5
**C14**	10:1	13.1	33.7	12.8	40.4
	40:1	7.80	19.8	15.7	56.6
**C16**	10:1	13.3	29.5	11.7	45.6
	40:1	12.1	27.7	11.5	48.6

Continue table S1 Content changes of α-glucosidase secondary structure under various concentrations of 16 AR-related flavonoids

NO.	Ratio*	α-helix (%)	β-sheet (%)	β-turn (%)	random coil (%)
**C17**	10:1	14.6	24.3	11.7	49.4
	40:1	17.4	31.3	13.6	37.8
**C19**	10:1	22.6	26.7	10.7	39.9
	40:1	43.2	6.70	10.2	39.9
**C28**	10:1	9.30	25.6	14.4	50.7
	40:1	10.9	20.7	12.9	55.5
**C29**	10:1	10.9	23.5	14.5	51.1
	40:1	8.50	23.4	13.2	55.0

*The molar ratios of the 16 flavonoids to α-glucosidase (4 μM).

Table S2 Binding energies and binding sites of 16 AR-related flavonoid compounds on α-glucosidase

NO.	Binding Energies （kcal/mol）	Hydrogen Bonding	Hydrophobic Interactions	π–π stacking	Salt Bridge	Cation–π Interactions
**C1**	-6.97	Glu304, Arg312, Arg439	Phe177, Thr215, Phe300, Asp349			
**C2**	-6.48	Asp68, Arg212, Glu276	Phe157, Phe158, Phe177	Tyr71, Phe158		
**C3**	-6.91	Asp214, Arg312, Asp349	Phe177, Thr215, Asp349, Arg439			
**C4**	-7.26	Asp68, His111, Arg212, Glu304, His348	Tyr71, Phe177, Thr215, Phe300, Asp349			
**C5**	-6.76	Asp68, Phe157, Arg212, Glu276	Phe157, Phe158, Phe177	Tyr71		
**C6**	-4.72	Asp68, Arg212, Glu276, Arg312, Try313	Tyr71, Phe158, Phe177, Phe298, Phe300, Tyr344, Arg439			
**C7**	-5.90	Glu276, Glu304, Arg439	Phe157, Phe158	Phe157		
**C8**	-6.84	Asp68, Arg212, Glu276, Glu304	Phe158, Phe177, Asp349	Tyr71, Phe300		Arg439
**C10**	-6.61	Asp68, Arg212, Glu276, Gln350, Asp408	Phe157, Phe158, Phe177	Tyr71		
**C11**	-6.70	Phe157, Glu276	Phe157, Phe158, Phe177, Thr215, Leu218, Glu276, Ala278			
**C14**	-7.10	Asp68, His111, Gln350, Asp408	Tyr71, Phe157, Phe158, Phe177			Arg439

Continue table S2 Binding energies and binding sites of 16 AR-related flavonoid compounds on *α*-glucosidase

NO.	Binding Energies（kcal/mol）	Hydrogen Bonding	Hydrophobic Interactions	π–π stacking	Salt Bridge	Cation–π Interactions
**C16**	-7.19	Asp68, Glu276, Arg312, His348, Asp349	Phe157, Thr215	Phe300		
**C17**	-6.95	His111, Asp214, Glu276	Tyr71, Phe177, Thr215	Phe300	Arg439	
**C19**	-5.14	Asp214, His348, Asp349	Phe158, Arg312			
**C28**	-6.74	Asp214, Glu304, Arg439	Tyr71, Phe177, Thr215, Phe300, Asp349	Tyr71		
**C29**	-6.82	Asp214, Arg312, Try313	Phe158, Thr215, Arg439	Phe177		
**Acar**	-6.63	Asp 214, Glu 276, Arg 312, Asp 408, Arg 439, Arg 443	Tyr 71, Phe 157		His 239, Arg 439	

Table S3 Effects of 15 AR-related flavonoid compounds on postprandial blood glucose levels in normal mice following oral sucrose administration

NO.	Dose (mg/kg)	Blood glucose levels	Rate of blood glucose reduction%
Model	-	14.58 ± 1.41	-
**C1** quercetin	10	14.46 ± 1.06	0.77
	50	13.41 ± 0.41	7.98
	100	12.26 ± 1.65**	15.87
Acarbose	20	7.39 ± 0.70****	49.31
Model	-	13.94 ± 0.77	-
**C2** kaempferol	10	12.68 ± 1.47	9.06
	50	12.79 ± 0.81	8.25
	100	11.88 ± 1.62*	14.80
Acarbose	20	7.38 ± 0.95****	47.09
Model	-	13.94 ± 0.77	-
**C3** liquiritigenin	10	14.06 ± 1.45	-0.90
	50	13.19 ± 1.35	5.38
	100	11.58 ± 1.23**	16.95
Acarbose	20	7.38 ± 0.95****	47.09
Model	-	13.94 ± 0.77	-
**C4** isoliquiritigenin	10	12.81 ± 1.00	8.07
	50	13.05 ± 1.44	6.37
	250	11.90 ± 0.62**	14.62
Acarbose	20	7.38 ± 0.95****	47.09
Model	-	15.44 ± 0.96	-
**C5** naringenin	5	14.56 ± 1.96	5.67
	25	13.23 ± 0.63**	14.33
	100	12.39 ± 1.03****	19.76
Acarbose	20	7.90 ± 0.56****	48.83

Continue table S3 Effects of 15 AR-related flavonoid compounds on postprandial blood glucose levels in normal mice following oral sucrose administration

NO.	Dose (mg/kg)	Blood glucose levels	Rate of blood glucose reduction%
Model	-	14.48 ± 0.88	-
**C6** rutin	2	14.68 ± 1.00	-1.38
	10	12.81 ± 1.23**	11.49
	50	11.83 ± 0.71****	18.31
Acarbose	20	8.09 ± 0.60****	44.13
Model	-	14.58 ± 1.41	-
**C8** genistein	25	13.91 ± 1.00	4.55
	50	13.26 ± 0.72	9.01
	100	12.06 ± 0.82****	17.24
Acarbose	20	7.39 ± 0.70****	49.31
Model	-	13.44 ± 2.01	-
**C10** isoquercitrin	10	12.41 ± 2.07	7.66
	25	12.21 ± 2.08	9.15
	50	10.91 ± 1.78*	18.82
Acarbose	20	7.16 ± 0.52****	46.72
Model		14.48 ± 0.88	
**C11** daidzein	10	13.55 ± 0.72	6.39
	25	13.13 ± 0.22**	9.33
	50	12.80 ± 0.93***	11.57
Acarbose	20	8.09 ± 0.60****	44.13
Model	-	14.48 ± 0.88	-
**C14** isorhamnetin-3-*O*-glucoside	10	14.84 ± 0.62	-2.50
	50	12.66 ± 0.48****	12.52
	100	12.19 ± 0.81****	15.80
Acarbose	20	8.09 ± 0.60****	44.13

Continue table S3 Effects of 15 AR-related flavonoid compounds on postprandial blood glucose levels in normal mice following oral sucrose administration

NO.	Dose (mg/kg)	Blood glucose levels	Rate of blood glucose reduction%
Model		15.44 ± 0.96	
	10	15.01 ± 1.15	2.75
**C16** calycosin-7-*O*-glucoside	25	13.71 ± 0.77**	11.17
	50	12.78 ± 0.74****	17.25
Acarbose	20	7.90 ± 0.56****	48.83
Model	-	15.44 ± 0.96	-
	10	14.46 ± 0.69	6.32
**C17** isomucronulatol	50	14.29 ± 0.80	7.45
	100	13.24 ± 1.18***	14.25
Acarbose	20	7.90 ± 0.56****	48.83
Model	-	15.44 ± 0.96	-
**C19** astrapterocarpan-3-*O*-glucoside	1	14.30 ± 1.16	7.37
	10	13.71 ± 0.78**	11.17
	100	13.31 ± 0.58***	13.77
Acarbose	20	7.90 ± 0.56****	48.83
Model	-	14.58 ± 1.41	-
	2	14.84 ± 0.62	-1.80
**C28** dihydrodaidzein	10	12.74 ± 0.75*	12.61
	50	11.39 ± 1.48****	21.87
Acarbose	20	7.39 ± 0.70****	49.31
Model	-	16.01 ± 0.68	-
**C29** equol	10	14.70 ± 1.36	8.20
	50	13.93 ± 0.62**	13.04
	100	12.56 ± 1.87****	21.55
Acarbose	20	7.98 ± 0.58****	50.02

Data are presented as mean ± SD (n=8), $^{*}P < 0.05$, $^{**}P < 0.01$, $^{***}P < 0.001$, and $^{****}P < 0.0001$, compared with the model group by ANOVA.

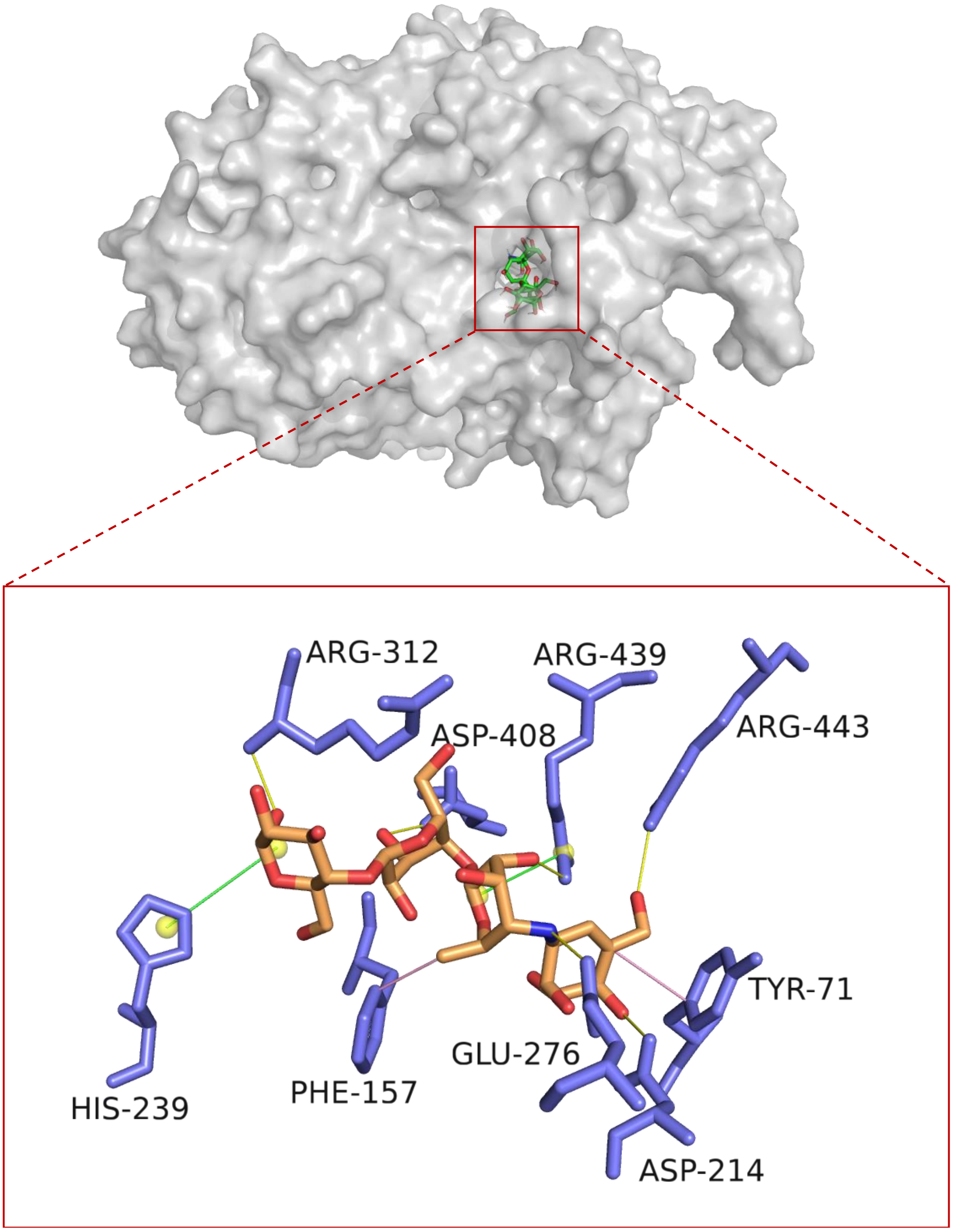

Figure S1 Molecular docking results of acarbose within the active pocket of α-glucosidase. Yellow lines indicate hydrogen bonds, green lines represent salt bridges, and pink lines indicate hydrophobic interactions.

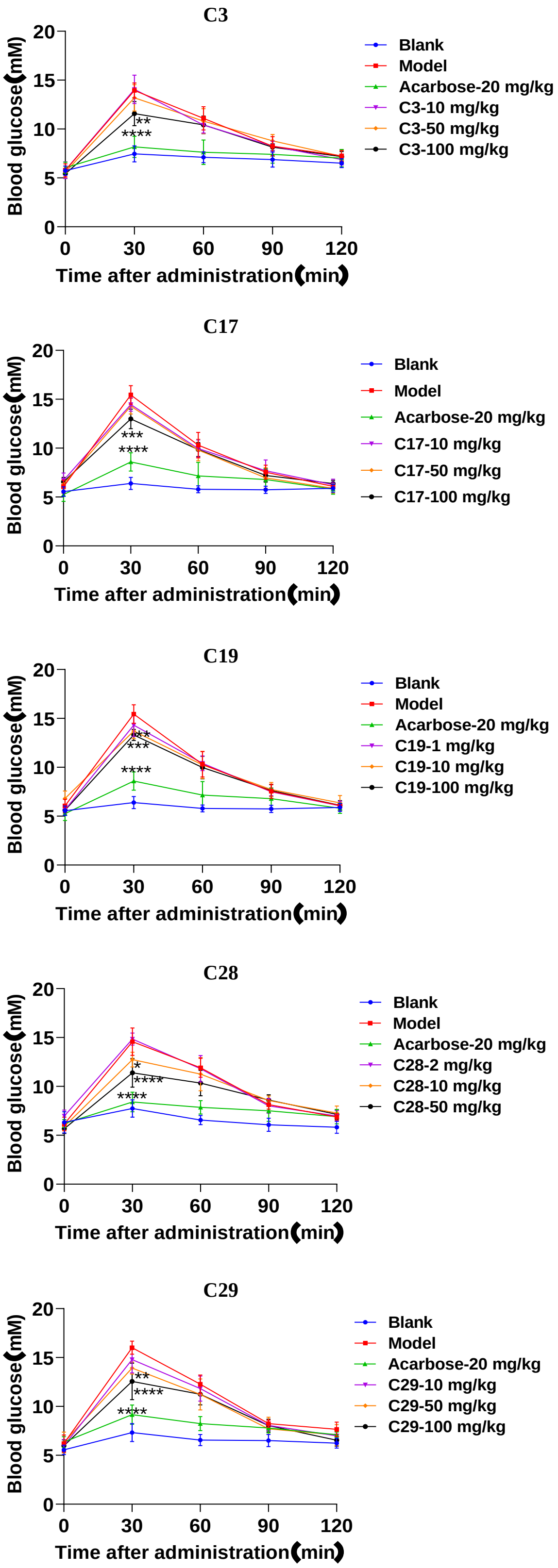

Figure S2 The effect of five newly identified flavonoids on postprandial blood glucose in mice at different time points. Significantly different from the control value, $^{**}P< 0.05$, $^{**}P< 0.01$, $^{***}P< 0.001$, $^{****}P< 0.0001$.
